# Anaphoric Reference to Quantified Expressions in Swedish

**DOI:** 10.1007/s10936-018-9618-z

**Published:** 2018-12-24

**Authors:** Fredrik Heinat, Eva Klingvall

**Affiliations:** 1 Department of Languages, Linnæus University, Växjö, Sweden; 2 Centre for Languages and Literature, Lund University, Lund, Sweden

**Keywords:** Semantics, Plausibility, Set focus, Quantifier size

## Abstract

This paper presents the results from two studies on anaphoric reference to quantifying expressions (QEs) in Swedish, contributing to the current cross-linguistic discussion on this issue. For English it has been shown that the polarity of the QE (positive vs negative) determines the anaphoric set reference (to the referens set, REFSET, or to the complement set, COMPSET), while for Spanish it has been claimed that while REFSET interpretation is the default, the relative sizes of the two sets (REFSET and COMPSET) also matters. In Experiment 1, a semantic plausibility study. The results showed that for positive QEs, anaphoric reference can only be to the REFSET, while for negative QEs, it can only be to the COMPSET. Unlike in English and Spanish, REFSET continuations were categorically ruled out for negative QEs. To investigate whether the internal differences between QEs could be explained in terms of set size, we conducted Experiment 2, an estimation task. The results from this experiment showed that the size of the REFSET relative to the COMPSET was not a determining factor.

## Introduction

According to Gundel et al. (1993), the form of a referring expression is determined by the cognitive status of the referent. Anaphoric reference by means of a pronoun, as in (1), signals that the expression referred to has the highest possible cognitive status; it is in focus (ibid.):^1^

**Table:** 

(1)	$\hbox {Mary}_i$ bought a new laptop and $\hbox {she}_i$ was very pleased

Unlike referring expressions, quantifying expressions (QEs) do not pick out specific referents but they specify the number or the proportion of entities for which some property holds. The entities for which the property holds are generally the attentional focus of the sentence (henceforth referred to as ‘focus’).^2^ In the first part of the sentence in (2), it is mentioned that there are two girls in a group. Intuitively one would expect that any of the two girls could be in focus. The anaphor *she* can however only pick out the individual for which the property of missing the bus holds.

**Table:** 

(2)	One of the two girls missed the bus and she was sorry
	*she* = the girl who missed the bus; *she*$\ne$ the girl who didn’t miss the bus

For other types of QEs establishing reference is not so straightforward.

**Table:** 

(3)	Some researchers went to the party

The sentence in (3) states something about researchers in relation to a party, more specifically that a number of members from the set of researchers attended a specific party. Two other groups of people might also be taken into account when interpreting the sentence: the group of potential researchers who didn’t attend the party and the potential group of party-goers who were not researchers (Sanford et al. 1996). These different groups of people are captured in the following Venn diagram:

**Fig. 1:**
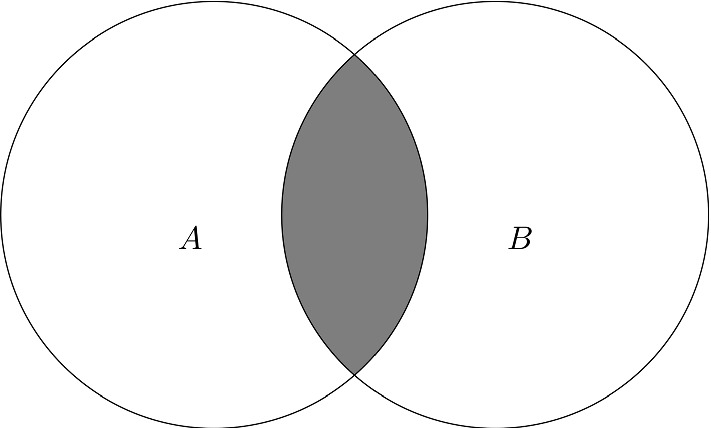
A: researchers B: people attending the party A$\cap$B: researchers attending the party A-B: researchers not attending the party B-A: non-researchers attending the party

Set A in Fig. 1 represents all researchers, while Set B represents people attending the party. The grey area, i.e. the intersection between Set A and Set B, represents the researchers who went to the party. Following Moxey and Sanford (1987), we call this set the reference set (refset). The part of Set A that is not in set B, i.e. the white area of Set A, is researchers for which the property of going to the party does not hold. We will call this subpart of Set A the complement set (compset) (Moxey and Sanford 1987).

Depending on what type of QE is used, different subparts of Set A can be in focus. We can see this when an anaphor is used to refer back to different types of QE, as in (4) and (5) below. *Some*, in (4), is a positive (upward entailing) quantifier, while *few*, in (5), is a negative (downward entailing) quantifier (Peters and Westerståhl 2006). Different parts of the set are in focus in the two cases.

**Table:** 

(4)	Some researcher went to the party and they were happy to be there

We naturally interpret the anaphor *they* in (4) as referring to the researchers who attended the party. In other words, *they* refers to the reference set in Fig. 1, the entities for which the statement in the preceding clause is true. That is not the case in (5), however:

**Table:** 

(5)	Few researcher went to the party and they were sad to have missed out

In (5), the anaphor *they* is most naturally interpreted as referring *not* to the researchers who went to the party, as in (4), but to the researchers who *didn’t* attend the party, i.e. the complement set in Fig. 1. Crucially, the latter ones are not even mentioned in the first part of the sentence but they can still be referred to by means of a pronoun, indicating that this subset of A is in focus in Gundel et al.’s terms (1993). For a quantifier like *few*, the anaphoric reference in (5) is not the only possibility as the anaphor could in fact also refer to the researchers attending the party, (6a). For a quantifier like *some*, this dual possibility does not exist as the anaphor can’t be interpreted as referring to the researchers not attending the party, (6b):

**Table:** 

(6)	a.	Few researcher went to the party and they were happy to be there
	b.	# Some researcher went to the party and they were sad to have missed it

*Some* and *one of the two*, in (2) above, thus behave in the same way, while *few* is different.

Moxey and Sanford (1987) have shown that speakers of English prefer anaphors following positive QEs to refer to the refset (as in (4)) and anaphors following negative QEs to refer to the compset (as in (5)), although the refset is also a possibility in the latter case (see (6a)). QEs are known to differ in a number of respects across languages (see e.g. Nouwen 2010; Tsai et al. 2014). In an introspective study, Zulaica-Hernández (2018) reports patterns for Spanish similar to those in English. However, he claims that a crucial factor is also the relative sizes of refset and compset counted in number of members. This is not the case for English, where relative set size does not determine set reference (Sanford et al. 1996). The difference between English and Spanish calls for further investigation of this issue in other languages. In this paper, we therefore investigate the role of polarity and relative set size in anaphoric reference to QEs in Swedish. In the next section, we give an overview of previous studies on anaphoric set reference. After that, we report the results from two experiments that we have conducted. In Experiment 1, we examine the role of polarity in anaphoric set reference in Swedish in a semantic plausibility study. In Experiment 2, we investigate the difference in proportion between different QEs to see whether the relative refset/compset size plays a role when determining anaphoric reference. We end the paper with a general discussion of the results and their implications for our understanding of set reference.

## Anaphoric Set Reference in English and Spanish

One thing that matters for the interpretation of anaphors is whether the quantifier is positive or negative, as mentioned above. Positive and negative quantifiers differ in what type of polarity item they can appear with and in their entailment patterns. Negative quantifiers, also called downward entailing or downwards monotone, can appear with negative polarity items, such as *anymore* in (7a), while positive quantifiers, also known as upward entailing or upwards monotone, cannot, (7b):

**Table:** 

(7)	a.	Not many researchers go to parties anymore
	b.	Some researchers go to parties (*anymore)

When it comes to entailment patterns, positive and negative quantifiers also have opposite patterns: for positive quantifiers, there is entailment ($\rightarrow$) from a subset (‘early party goers’) to the superset (‘party goers’), (8a), while for negative quantifiers, there is entailment from the superset to a subset, (8b):

**Table:** 

(8)	a.	Some researchers went to the party early $\rightarrow$ Some researchers went to the party
	b.	Not many researchers went to the party $\rightarrow$ Not many researchers went to the party early

In a series of experiments, L. Moxey and A. Sanford (and colleagues) have investigated anaphoric reference to QEs in English. The pattern emerging is that positive QEs virtually only allow for anaphoric expressions to point to the refset, while negative QEs allow anaphoric expressions to point either to the refset or the compset. A considerable number of positive and negative QEs were investigated in four studies reported in Moxey and Sanford (1987) and Sanford et al. (1996). Participants were asked to write down continuations of sentences as in (9), where either a positive quantifier like *a few* or a negative quantifier like *few* was present:

**Table:** 

(9)	A few/Few many MPs went to the meeting. They ...

Positive QEs were almost always followed by clauses where *they* referred to the refset (90% of the cases, vs. 0.5% cases with compset). For negative QEs, there was more variation but in the majority of cases, the anaphoric expression pointed to the compset rather than the refset (62% with compset vs. 21% with refset).

Moxey et al. (2001) and Moxey (2006) add some finer details to the anaphoric reference patterns for QEs. Moxey et al. (2001) show that a small number of negative QEs are less likely to be followed by anaphoric reference to the compset. According to the authors, negative QEs are in general like ordinary negation in involving the denial of a supposition. A sentence like *John didn’t go to the cinema* is most informative if there was an expectation that John would go to the cinema. Sentences with negative QEs are similar. A sentence like *few people attended the meeting* thus comes with a supposition that more people were expected to attend the meeting (2001, 430). Negative QEs, thus generally both state the proportion of X for which some property holds, and imply that more X were expected to have this property. Unlike most other negative QEs, negative QEs of the type *at most 10%* are exceptional, however, in that they do not deny a supposition.^3^ Although such negative QEs were less likely to be followed by compset continuations in the study, compset continuations were still possible. Moxey (2006) shows, more generally, that the likelihood of producing a compset continuation can also be dependent on explicitly stated expectations: if a positive QE denotes a quantity that is smaller than an explicitly stated expectation, anaphoric reference to the compset is possible. refset continuations are however still by far more likely even in such contexts. Similarly, a negative QE is less likely to be followed by a compset continuation when the expected quantity is zero than when the expectation is higher. However, although the manipulated context affected the reference patterns for negative QEs in Moxey’s study, these were still followed by compset continuation in half of the cases for some negative QEs and in a vast majority of the cases for other negative QEs.

In an introspective study of Spanish, Zulaica-Hernández (2018) confirms that the general pattern identified for English holds also for Spanish: positive quantifiers can only be followed by anaphors referring back to the refset, while for negative quantifiers anaphoric reference to either the refset or the compset is possible. According to Zulaica-Hernández , two principles are at play. Firstly, refset interpretation for anaphors is the default case irrespective of the polarity of the QE. Secondly, the set with the largest number of members is most easily referred to. Zulaica-Hernández claims that the two principles coincide for positive QEs, but not for negative ones. That is, for negative but not positive QEs, the compset is often bigger than the refset. Therefore, negative QEs allow for anaphors to pick out either the compset or the refset. These proposed principles do not seem to hold in English. In a pre-study for one of their continuation tasks, Sanford et al. (1996) asked participants to estimate the proportion denoted by a number of QEs. The negative QEs *few* and *not quite all* were estimated to denote 13% and 95% respectively, but appeared with virtually the same number of compset continuations, 20 and 19, respectively. Furthermore, the positive QE *a few* was estimated to denote 15%, which is approximately the same proportion as *few* (13%), and thus picked out a relatively small refset. It was nevertheless never followed by compset continuations.

Since the findings in the studies on anaphoric reference to QEs in English (polarity determines set reference) and the principles claimed to hold for Spanish (default refset reference but modulated by set size) are not the same, questions arise whether these three properties determine anaphoric reference to QEs in Swedish. In the following, we report the results from two experiments on Swedish. In Experiment 1, we investigated what anaphoric set reference positive and negative QEs allow for in Swedish and in Experiment 2, we investigated the relative proportion of refset and compset for a number of QEs in Swedish to see if there were correlations between refset/compset size and anaphoric reference patterns.

## Experiment 1: A Semantic Plausibility Study

Since there are no previous studies on the focus properties of QEs in Swedish, Experiment 1 is designed to answer the very general question if there are preferred patterns for anaphoric reference to positive and negative QEs. The QEs we employed in the experiment are a selection of Swedish counterparts of the QEs investigated in Moxey and Sanford (1987) and Filik et al. (2011). However, the method is slightly different from the continuation tasks in various work by Moxey, Sandford and colleagues (see above). In order to find out the focus properties of QEs in Swedish, we conducted a semantic plausibility study.

### Method

#### Participants

44 native speakers of Swedish, all undergraduate students at Lund University or Linnæus University, took part in the study in exchange for one cinema ticket each.

#### Materials

168 test items were created, each of which contained four sentences manipulated along two dimensions; Quantifier (pos vs. neg) and Set Reference of the disambiguating adjective (refset vs. compset), as in (10) (in boldface here for expository reasons):

**Table:** 

(10)	a.	**Nästanalla** ekonomer hade koll på budgeten och att almost all finance officers were up to date with budget-def and that de var så **oinsatta** märktes vid bokslutet. [pos–refset] they were so informed noticed-pass in annual report-def ‘Almost all finance officers were up to date with the budget and that they were so well-informed was noticeable in the annual report’
	b.	**Nästanalla** ekonomer hade koll på budgeten och att almost all finance officers were up to date with budget-def and that de var så **oinsatta** märktes vid bokslutet. [pos–compset] they were so uninformed noticed-pass in annual report-def ‘Almost all finance officers were up to date with the budget and that they were so uninformed was noticeable in the annual report’
	c.	**Intealla** ekonomer hade koll på budgeten och att de not all finance officers were up to date with budget-def and that they var så **insatta** märktes vid bokslutet. [neg–refset] were so informed noticed-pass in annual report-def ‘Not all finance officers were up to date with the budget and that they were so well-informed was noticeable in the annual report’
	d.	**Intealla** ekonomer hade koll på budgeten och att de not all finance officers were up to date with budget-def and that they var så **oinsatta** märktes vid bokslutet. [neg–compset] were so uninformed noticed-pass in annual report-def ‘Not all finance officers were up to date with the budget and that they were so uninformed was noticeable in the annual report’

The QEs included were the positive QEs: *några* (‘some’), *många* (‘many’), *nästan alla* (‘almost all’), and *alla* (‘all’); and the negative QEs: *få* (‘few’), *inte många* (‘not many’), *inte alla’* (‘not all’), and *inga* (‘no’). The polarity of the QEs is based on their entailment behaviour, as described above.

The experimental items were distributed across four lists in a Latin square design. Each list appeared in two versions with inverted item orders. Each participant only read one sentence from each item, but read all types of manipulation.

#### Procedure

The experimental items were implemented in Google Docs and were presented one by one on a computer screen. Each sentence was followed by a question as to whether the sentence sounded ‘good’, ‘bad’ or ‘in between’ (*bra/dålig/sådär*).^4^ A rating was required before the participant could proceed to the next sentence and it was not possible to go back and change the rating. There were two experiment sessions, one at Lund University and one at Linnæus University, and both were conducted in computer labs.

#### Statistical Analysis

The statistical analysis was made using R (R Core Team 2018) and linear mixed effects models using the package lmerTest (Kuznetsova et al. 2017). When analyzing the results, it became obvious that the ‘bad’ and ‘in between’ ratings were sometimes dependent on factors other than set reference. For this reason, the category ‘in between’ was conflated with the ‘bad’ category. The statistical models presented in this section thus focus on percentages of ‘good’ ratings.

### Results

**Fig. 2:**
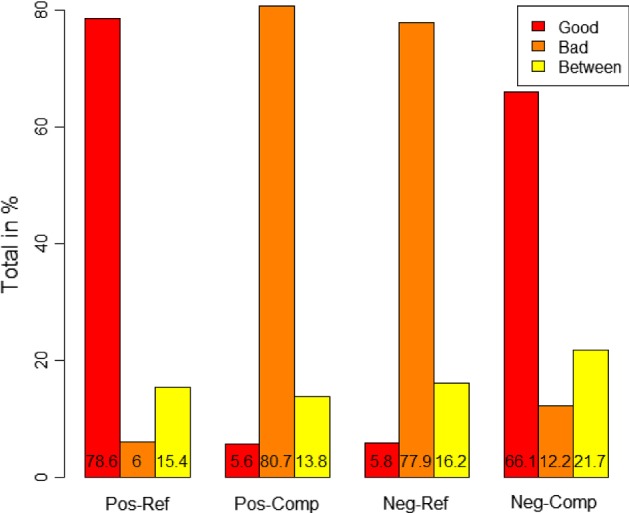
Ratings of QEs

As seen in Fig. 2, the results were that positive quantifiers with anaphoric reference to the refset were judged as semantically congruent, while they were judged as anomalous with anaphoric reference to the compset. We also see that for the negative quantifiers, the opposite pattern emerged: they were judged as congruent with anaphoric reference to the compset but anomalous with the reference to the refset.

**Table 1:** Results from Exp 1. Positive and negative QEs

	Estimate	SE	df	*t* Value	Pr($>|t|$)
Positive QE–ref.set (intercept)	0.78451	0.01727	140.30000	45.42	< 2e−16***
Negative QE–ref.set	− 0.72662	0.01744	666.00000	− 41.67	< 2e−16***
Positive QE–comp.set	− 0.72917	0.01744	666.00000	− 41.81	< 2e−16***
Negative QE–comp.set	1.33034	0.02466	666.00000	53.95	< 2e−16***

Signif. codes: 0 ‘***’ 0.001 ‘**’ 0.01 ‘*’ 0.05 ‘.’

Model: Grade $\sim$ Polarity * Set + (1 + Polarity | Participant) + (1 + Polarity | Item)

From Table 1, we see that all the differences between the conditions are significant. It is clear both that the refset is the focussed set in anaphoric reference to positive QEs and that the compset is the focussed set in anaphoric reference to negative QEs (a relevelling of the model in Table 1 shows this). However, there is a significant difference between these two ratings. The sentences with positive QEs and refset continuations are rated as ‘good’ to a significantly higher degree (appr 0.12) than sentences with negative QEs and compset continuations. A relevelling of the results in Table 1 shows that there is also a very small (0.002), but significant difference between the ratings of positive QEs with compset continuations and negative QEs with refset continuations.

**Fig. 3:**
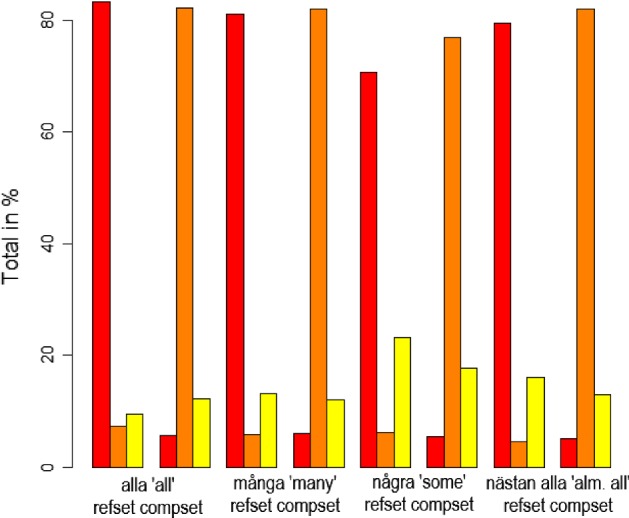
Ratings of positive QEs

There were also internal differences within the groups of positive and negative quantifiers. We start by looking at the positive QEs. As can be seen in Fig. 3, the QE *några* (‘some’) differed from the other QEs in the refset condition. The results from the statistical analysis confirm this: Table 2 shows that *några* (‘some’) was significantly different from the other positive quantifiers when reference was made to the refset (a relevelling indicates that there are no other significant differences).^5^

**Table 2:** Results from Exp 1. Positive QEs—refset

	Estimate	SE	df	*t* Value	Pr($>|t|$)
*några* (intercept)	0.69350	0.02785	451.10000	24.906	< 2e−16***
*nästan alla*	0.10569	0.03600	684.50000	2.936	0.00344**
*alla*	0.14475	0.03580	682.30000	4.044	5.86e−05***
*många*	0.11471	0.03601	684.60000	3.186	0.00151**

Signif. codes: 0 ‘***’ 0.001 ‘**’ 0.01 ‘*’ 0.05 ‘.’

Model: Grade $\sim$ Quantifier * Set + (1 | Participant) + (1 | Item)

A relevelling of the factors in the model shows that there were no significant differences between the positive QEs in the compset condition.

**Fig. 4:**
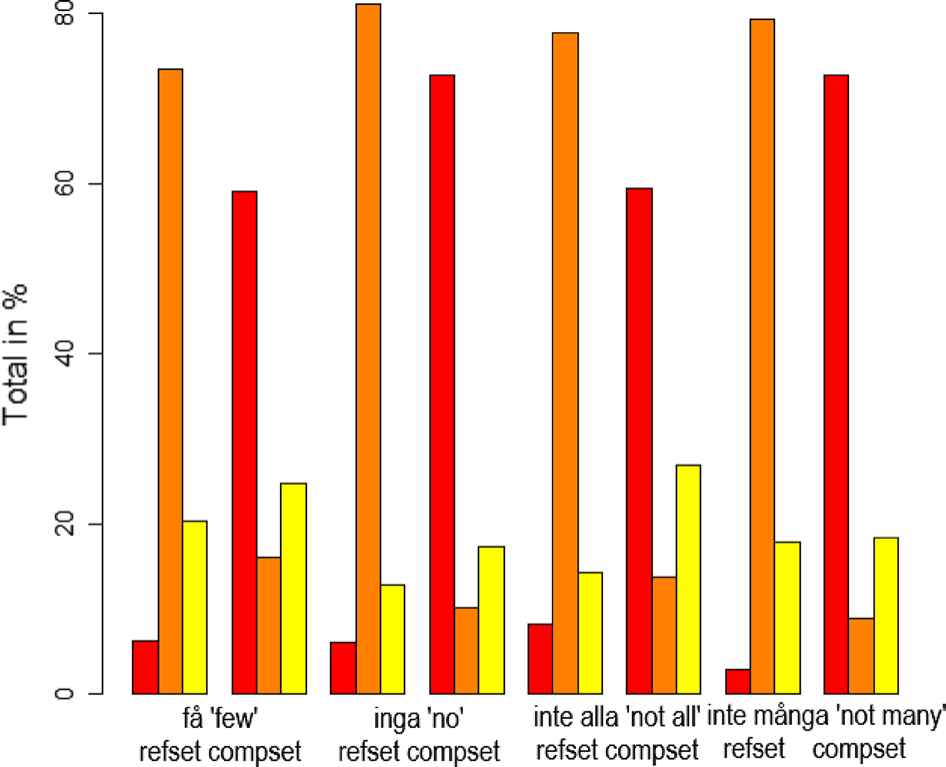
Ratings of negative QEs

Turning to the negative QEs, shown in Fig. 4, we again find differences. The QEs *få* (‘few’) and *inte alla* (‘not all’) were judged as semantically congruent to a lesser degree than the other two negative QEs when reference was made to the compset, as shown in Table 3. A relevelling of the factors shows that *inte alla* differs significantly from *inte många* (‘not many’) and *inga* (‘no’).

**Table 3:** Results from Exp 1. Negative QEs—compset

	Estimate	SE	df	*t* Value	Pr($>|t|$)
*få* (Intercept)	0.59434	0.02785	451.10000	21.344	< 2e−16***
*inga*	0.13992	0.03580	682.30000	3.909	0.000102***
*inte alla*	− 0.01187	0.03601	684.70000	− 0.329	0.741885
*inte många*	0.13136	0.03600	684.40000	3.649	0.000283***

Signif. codes: 0 ‘***’ 0.001 ‘**’ 0.01 ‘*’ 0.05 ‘.’

Model: Grade $\sim$ Quantifier * Set + (1 | Participant) + (1 | Item)

In contrast to the positive QEs, there were differences in the less preferred anaphoric condition for negative QEs, i.e the refset condition. As shown in Table 4, there was a significant difference between the two QEs *inte alla* (‘not all’) and *inte många* (‘not many’). Anaphoric reference to the refset was graded as slightly better for *inte alla* than for *inte många*. There were no other significant differences between the negative QEs in this condition.

**Table 4:** Results from Exp 1. Negative QEs – refset

	Estimate	SE	df	*t* Value	Pr($>|t|$)
*inte alla* (intercept)	0.09101	0.02626	275.60000	3.465	0.000614***
*få*	− 0.02689	0.03369	357.10000	− 0.798	0.425339
*inte många*	− 0.06954	0.03382	357.40000	− 2.056	0.040489*
*inga*	− 0.03646	0.03395	358.20000	− 1.074	0.283577

Signif. codes: 0 ‘***’ 0.001 ‘**’ 0.01 ‘*’ 0.05 ‘.’

Model: Grade $\sim$ Quantifier * Set + (1 | Participant) + (1 | Item)

Before discussing the results from Experiment 1, we will give a summary of the results. The first four bullets points below pertain to the QEs divided into two categories: positive and negative. The last three bullets points deal with individual QEs.

- Positive QEs were graded as ‘good’ to a higher degree with refset continuations than with compset continuations.
- Negative QEs were graded as ‘good’ to a higher degree with compset continuations than with refset continuations.
- Positive QEs with refset continuations were graded as ‘good’ to a higher degree than negative QEs with compset continuations.
- Negative QEs with refset continuations were graded as ‘good’ to a higher degree than positive QEs with compset continuations.
- The positive QE *några* (‘some’) was graded as ‘good’ to a lesser degree than the other positive QEs with refset continuation.
- The negative QEs *få* (‘few’) and *inte alla* (‘not all’) were graded as ‘good’ to a lesser degree than the other negative QEs with compset continuation.
- The negative QE *inte alla* (‘not all’) was graded as ‘good’ to a higher degree than the negative QE *inte många* (‘not many’) with refset continuations.

### Discussion

The first experiment was a semantic plausibility study. It was designed to address the question wether Swedish QEs give rise to focussing effects in the way QEs do in English and Spanish. The experiment addressed the question if any effects are related to the polarity of the QEs.

The results showed that Swedish QEs indeed give rise to focussing effects, and that these seem to be related to the polarity of the QEs: All positive QEs favoured refset continuations, and all negative QEs favoured compset continuations. These results pattern with results from previous studies on English (see e.g. Moxey and Sanford 1987; Sanford et al. 1996) and Spanish (Zulaica-Hernández 2018). The finding that refset continuations were dispreferred to such extent following negative QEs is surprising though. Recall that Sanford et al. (1996) had 21% refset continuations following negative QEs. Our results show quite clearly that refset is not the default interpretation for negative QEs in Swedish and, given the low ratings it is even questionable whether it is always possible, as claimed for English (Nouwen 2003) and Spanish (Zulaica-Hernández 2018). It should be noted, however, that all three studies use different methods.

Furthermore, there was also a significant difference between the positive and negative QEs with their favoured continuations, refset and compset, respectively. The positive QEs were rated as ‘good’ to a higher degree (approx. 12 percentage points) than the negative QEs. Moxey, Sanford and colleagues show that negative QEs in English can be followed either by refset or compset continuations. At first glance, one might think that the reason that the negative QEs in the compset condition in our experiment show slightly worse ratings than the positive QEs in the refset is their possibility to focus the refset. However, if this was the reason, we would expect there to be a difference between the negative QEs with refset continuations and the positive QEs with compset continuations. There is a significant difference between these two types of sentences in the expected direction, but the size of the difference (approx. 0.2 percent units) is much too small to support this explanation. We interpret this very small difference to be a spurious effect, and not something that can account for the fact that negative QEs with compset continuations are not rated as good as positive QEs with refset continuations. Instead, we suspect that individual QEs, or some experimental items, might be behind this result.

Before we discuss the individual QEs, it should be noted that the experimental set up was not to compare the individual QEs to each other. Each item contrasts one positive QE with one negative QE. Moxey and Sanford (1987, among others) note that context can have an influence on the interpretation of the anaphoric element. Even the sentence where the QE appears can exert contextual influence. Since QEs with the same polarity never appear in the same item in our experiment, differences between QEs with the same polarity could be due to the particular context induced by the sentence.^6^ However, with this in mind, we think that it is fruitful to look at the individual QEs to see if there are patterns that need to be pursued in future research.

What is noteworthy in the results is the internal variation within the groups of positive and negative QEs respectively. As mentioned above, the positive QE *några* (‘some’) and the negative QEs *få* (‘few’) and *inte alla* (‘not all’) stood out within their groups, as they gave rise to less uniform judgements than the other QEs. One might ask if at least part of the variation could be explained in terms of set size, as proposed by Zulaica-Hernández (2018). Looking at Spanish, Zulaica-Hernández claims that while the refset is always the default set for all QEs, they differ in how easily they appear with a compset depending on how large proportions they pick out. The larger the set, the easier it is to refer to it. Negative QEs picking out small refsets would then more easily appear with anaphoric reference to the compset than negative QEs picking out larger refsets, and the same for positive QEs. In his discussion, Zulaica-Hernández (2018, 460–461) assumes that in Spanish *all* negative QEs pick out bigger compsets than refsets, and that *all* positive QEs pick out bigger refsets than compsets. The question is whether this holds for Swedish too, and if the differences we find between individual QEs in Experiment 1 can be accounted for by the relative size of the set they pick out. This is what we investigated in Experiment 2.

## Experiment 2: Numerical Interpretation of QEs

In Experiment 2, we investigated the claim in Zulaica-Hernández (2018, 460–461) that the relative sizes of the refset and compset, in terms of number of members, influence anaphoric set interpretation. As mentioned above, Zulaica-Hernández (2018) claims that the set with the largest number of members is the one most easily referred to. As pointed out above, these principles do not hold for QEs in English. The question is if they hold in Swedish. In relation to the results from Experiment 1, the principle of largest set means that we expect all negative QEs to pick out a refset that is smaller than the compset. In addition, if Zulaica-Hernández’s claims about positive and negative QEs in Spanish (i.e that negative QEs pick out larger compsets and positive QEs pick out larger refsets) hold for Swedish QEs, we expect that the positive QEs should all pick out larger refsets than compsets.

### Method

#### Participants

210 native speakers of Swedish, all undergraduate students at Lund University or Linnæus University, took part in the study. Due to misunderstanding of the task, or illegible handwriting, the responses from 13 participants were excluded from the results.

#### Materials

Six experimental items were constructed differing only in what QE was used. The positive QEs included were: *många* (‘many’), *några* (‘some’) and *nästan alla* (‘almost all’). The negative QEs were: *få* (‘few’), *inte alla’* (‘not all’) and *inte många* (‘not many’). Neither *alla* (‘all’) nor *inga* (‘no’) was included since the compset and the refset, respectively, must logically be empty in these cases. Even though they were not included in the items, it should be noted that in Experiment 1 they both behave as expected according to the two principles discussed by Zulaica-Hernández (2018). Each item consisted of a context sentence stating the total number of set members, followed by a sentence containing one of the six QEs, and finally a question about the number of individuals for which the property holds, i.e members of the refset.

**Table:** 

(11)	Det var 100 studenter i den stora föreläsningslokalen. QE av dem hade varit där förut
	Hur många studenter hade varit där förut? (svara med siffror)
	(*There were 100 students in the auditory. QE of them had been there before*
	*How many do you think had been there before? (Give your answer in numbers)*)

Each participant saw only this single item and provided one single answer in line with a study in Moxey and Sanford (1993).

#### Procedure

The questionnaire was administered in connection with lectures. Oral instructions specifying that answers should be given in numbers were provided to make sure that the participants wrote precise numbers rather than relative sizes (such as ‘more/less than half’).

### Results

**Fig. 5:**
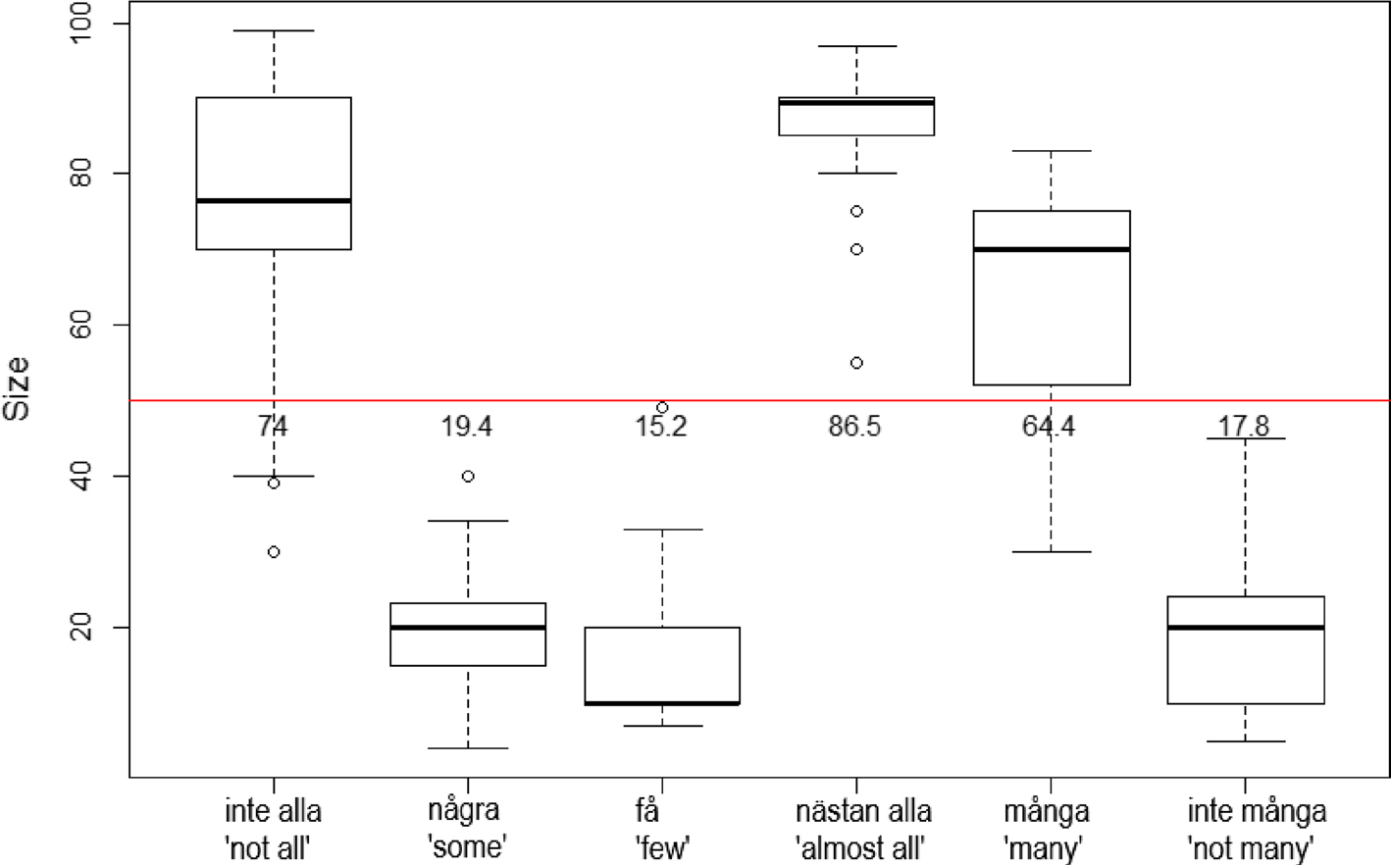
Estimation of QEs

After the exclusion of 13 participants (see above), all QEs had between 30 and 35 responses each. The boxplot in Fig. 5 shows the results from the questionnaire.

As seen in Fig. 5 the rating of the negative QE *inte alla* (‘not all’) was above 50 (the red horizontal line in the figure). This means that *inte alla* picked out a larger refset than compset, whereas the other two negative QEs picked out refsets that are smaller than the compsets. The two positive QEs *nästan alla* (‘almost all’) and *många* (‘many’) picked out refsets larger than the compsets, while *några* (‘some’) picked out a larger compset than refset.

When it comes to differences between the different QEs, a linear model reveals that there were significant differences between them. In Table 5, the positive QE *nästan alla* is the intercept and as can be seen, it picked out a significantly larger refset than all other QEs in the study.

In order to get an accessible overview of any other significant differences between the different QEs, the factors have been relevelled. In Table 6, the QE *några* is the intercept. What is notable here is that, in addition to picking out a significantly smaller refset than the other two positive QEs, there was no significant difference between *några* and two of the negative QEs, namely *få* (‘few’) and *inte många* (‘not many) when it comes to the size of the refset.

With the QE *få* as intercept, as in Table 7, it is clear that this QE picked out a significantly smaller refset than *inte alla*, and also that it did not differ significantly from the negative QE *inte många*.

Finally, when *inte alla* is the intercept, as in Table 8, we see that it differs significantly from all other quantifiers, including *inte många*.

**Table 5:** Results from Exp 2. Intercept *nästan alla*

	Estimate	SE	*t* Value	Pr($>|t|$)
*nästan alla* (intercept)	86.529	2.084	41.516	< 2e−16***
*få*	− 71.287	2.970	− 24.004	< 2e−16***
*många*	− 22.176	2.948	− 7.524	2.03e−12***
*inte alla*	− 12.563	3.044	− 4.127	5.49e−05***
*inte många*	− 68.729	2.926	− 23.486	< 2e−16***
*några*	− 67.142	3.018	− 22.247	< 2e−16***

Signif. codes: 0 ‘***’ 0.001 ‘**’ 0.01 ‘*’ 0.05 ‘.’

**Table 6:** Results from Exp 2. Intercept *några*

	Estimate	SE	*t* Value	Pr($>|t|$)
*några* (Intercept)	19.387	2.183	8.882	4.82e−16***
*få*	− 4.145	3.040	− 1.363	0.174
*nästan alla*	67.142	3.018	22.247	< 2e−16***
*många*	44.966	3.018	14.899	< 2e−16***
*inte alla*	54.580	3.113	17.536	< 2e−16***
*inte många*	− 1.587	2.997	− 0.529	0.597

Signif. codes: 0 ‘***’ 0.001 ‘**’ 0.01 ‘*’ 0.05 ‘.’

**Table 7:** Results from Exp 2. Intercept *få*

	Estimate	SE	*t* Value	Pr($>|t|$)
*få* (Intercept)	15.242	2.116	7.205	1.31e−11***
*nästan alla*	71.287	2.970	24.004	< 2e−16***
*många*	49.111	2.970	16.537	< 2e−16***
*inte alla*	58.724	3.066	19.155	< 2e−16***
*inte många*	2.558	2.949	0.867	0.387
*några*	4.145	3.040	1.363	0.174

Signif. codes: 0 ‘***’ 0.001 ‘**’ 0.01 ‘*’ 0.05 ‘.’

**Table 8:** Results from Exp 2. Intercept *inte alla*

	Estimate	SE	*t* Value	Pr($>|t|$)
*inte alla* (Intercept)	73.967	2.219	33.336	< 2e−16***
*några*	− 54.580	3.113	− 17.536	< 2e−16***
*få*	− 58.724	3.066	− 19.155	< 2e−16***
*nästan alla*	12.563	3.044	4.127	5.49e−05***
*många*	− 9.614	3.044	− 3.158	0.00185**
*inte många*	− 56.167	3.024	− 18.575	< 2e−16***

Signif. codes: 0 ‘***’ 0.001 ‘**’ 0.01 ‘*’ 0.05 ‘.’

### Discussion

The results from Experiment 2 are in line with those from Sanford et al. (1996). First, we found that for Swedish, it was not the case that all negative QEs picked out a bigger compset than refset, or that all positive QEs picked out a bigger refset than compset, in contrast to what Zulaica-Hernández (2018, 461) claims for Spanish. Second, we found that there were significant differences between QEs of the same polarity.

Interestingly, we also found that there was no significant difference in size between the positive and negative QEs that pick out a smaller refset than compset: the negative QEs *få* (‘few’) and *inte många* (‘not many’) picked out refsets of the same size as the positive QE *några* (‘some’). These findings do not correlate completely with those in Experiment 1, however. *Några* was graded as good to a lesser extent than the other positive QEs when the anaphoric reference targeted the refset. This is partly consistent with its set size (although refset continuations were still preferred over compset continuations), since *några* was the only positive QE that picked out a smaller refset than compset. However, if relative set size is important in determining set focus, the crucial difference between *några* and the other positive QEs should be in the compset condition. Since *några* picked out a bigger compset than refset, compset reference for *några* should be graded as ‘good’ to a higher extent than for the other positive QEs, which pick out bigger refsets than compsets. The results from Experiment 1 show that this is not the case: there was no significant difference between any of the positive QEs in the compset condition. We therefore draw the conclusion that relative set size does not influence the set focus of positive QEs in any obvious way.

Regarding the negative QEs in the compset condition in Experiment 1, we found that the QEs *få* (‘few’) and *inte alla* (‘not all’) were rated as ‘good’ to a lesser extent than the other two negative QEs. These findings do not seem to be explained in terms of set size either. While *inte alla* picked out a bigger refset than compset, potentially explaining the results in Experiment 1, *få* picked out a bigger compset than refset, leaving the results in Experiment 1 unexplained. In addition, although the negative QEs *inte många* (‘not many’) and *få* did not differ in set size, they differed in rating in Experiment 1. It therefore seems very unlikely that set size can explain the difference in rating in Experiment 1 between negative QEs with compset continuations.

In the sentences with negative QEs and refset continuations there was some indication that relative set size may influence set focus. In this experimental condition, the sentences with *inte alla* (‘not all’) were graded as ‘good’ more often than the sentences with *inte många* (‘not many’). This is consistent with the set size of *inte alla* since it picks out a bigger refset than compset. However, there were no other significant differences in this condition; not even *inga* (‘no’), which obviously picks out a bigger compset than refset, differed from *inte alla* in rating. We take this result to indicate that relative set size does not influence set focus with negative QEs either.

## General Discussion

The conclusions we draw from the results from the two experiments are the following; polarity determines what kind of anaphoric reference is made to a QE in Swedish.^7^ Positive QEs induce anaphoric reference to the refset and negative QEs induce anaphoric reference to the compset. This holds irrespective of the relative set size that the QE denotes. There is no evidence in our study that refset is ever the default interpretation for negative QEs in Swedish. Even more, the results from Experiment 1 seem to indicate that the refset is often not even an option for negative QEs in Swedish, in contrast to English and Spanish.

It is not entirely clear why we find individual differences between the QEs. As pointed out above, these individual differences cannot be accounted for by relative set size. The individual variation could in part be due to the nature of the task; the participants were asked to provide a judgement for the whole sentence. Sentences that were judged as ‘bad’ or ‘in between’ might get these judgements not because the anaphoric set reference was anomalous but for some other reason. To some degree, this is what must be going on in some unexpected judgements of the sentences containing the QEs *alla* (‘alla’) and *inga* (‘no’). In a few cases, sentences with anaphoric reference to the refset in the context of *alla* and to the compset in the context of *inga* were judged as ‘in between’ or ‘bad’, rather than ‘good’. Since it is logically impossible to refer to the compset in the context of *alla* and to the refset in the context of *no*, the perceived (partial) anomaly is likely to be due either to performance errors or to some other thing in the sentence. However, this is not likely to account for all of the variation within the groups of positive and negative QEs, respectively. At this point it is not clear what causes this variation and we can only offer some speculations that need to be investigated further. It has been suggested to us that some QEs might be dispreferred in subject position and that this affects the ratings for these particular QEs even when the anaphoric reference is to the preferred set. While a corpus investigation seems to indicate that the syntactic position is probably not what lies behind the lower ratings of some QEs,^8^ this issue should be investigated in more detail both in a more extensive corpus study and through judgement tests, where syntactic position is an experimental manipulation.

Another potential factor for the variance in Experiment 1 is pragmatic inference, maybe in combination with the syntactic factor of position. It might be the case that not all QEs are equally good sentence initially when an appropriate context is not already provided. A sentence like *not all students attended the lecture* might be considered infelicitous out of context, perhaps because negative QEs involve the denial of a supposition, as argued in Moxey et al. (2001) and Moxey (2006). It might thus be the case that certain negative QEs are less felicitous without the expectation being explicitly stated in the context. This too needs to be further investigated. We can compare this to the use of scalar adjectives. Even though the adjectives *long* and *short* occupy different edges of the same scale, only *long* can be used without any previous knowledge of the length of an object, as in *how long is the break?*. The use of *short* in the same question inevitably requires a context where it assumed that the break is short: *how short is the break?*.

Taken together, it is likely that different explanations will be required to account for the internal differences within the groups of positive and negative QEs, respectively. We might thus need to look at the individual QEs and their specific properties, instead of seeking a general explanation.

## Conclusion

We have reported the results from two experiments on anaphoric reference to quantified expressions in Swedish. In Experiment 1, where participants rated sentences on semantic plausibility, the conclusion is that the polarity of the QE determines what set is in focus and is referred to by an anaphoric pronoun. For positive QEs the refset is in focus and for negative QEs the compset is in focus. In Experiment 2, in which participants estimated the relative size of a QE’s refset and compset, the results showed that the relative size of the sets does not influence set focus in any obvious way, contrary to what has been claimed for Spanish (Zulaica-Hernández 2018). A more careful study involving an even distribution of both positive and negative ‘big’ and ‘small’ QEs might reveal more about relative set sizes and set focus, but currently it is still unclear why there are differences between some of the QEs, and we think that there are still many things left to be discovered about the set focus of QEs in Swedish, as well as cross-linguistically.

## Footnotes

1 Subscripts indicate co-reference.

2 The term ‘focus’ is always used to refer to ‘attentional focus’ in this article, and not to other types of focus.

3 Tag questions are used to check whether a quantifier comes with the denial of a supposition. Positive tag questions appear when a supposition is denied, while negative tag questions appear when there is no denial (Moxey et al. 2001). Quantifiers like *at most 10%* are negative in the sense that they are monotone decreasing, but they do not deny a supposition: *at most 10% of the students attended the lecture, didn’t they*.

4 We opted for three grades rather than just two, not to create ‘false’ good or bad ratings.

5 All results on individual quantifiers are from the same model: Grade $\sim$ Quantifier * Set + (1 | Participant) + (1 | Item), but the tables focus on different aspects, and are relevelled for expository reasons.

6 For instance, the negative QEs *få* (‘few’) and *inte alla* (‘not all’) never occur in the same item. Differences between them could thus be due to a difference in context induced by the sentences in which they occur.

7 It should be noted that there might be other factors that we have not controlled for in the study, for example context and expectation.

8 The corpus investigation was carried out using all subcorpora in *Språkbanken*. Since the corpora are not parsed the searches were restricted to QE + noun in sentence initial position as well as total number of occurrences of QE + noun. A search in 13.22 Gtoken on Nov. 2nd, 2018 gave the following frequencies of the QEs in sentence initial position compared to the total number of occurrences (absolute frequencies in parenthesis):

$$ \begin{array}{*{20}l}    {{\text{`few'  +  noun:}}} \hfill & {1.9\% } \hfill & {{\text{(sentence}}\,{\text{initial:}}\,\,{\text{10,447,}}\,\,{\text{total:}}\,\,{\text{545,241)}}} \hfill  \\    {{\text{`not many'  +  noun:}}} \hfill & {9.4\% } \hfill & {{\text{(sentence}}\,{\text{initial:}}\,\,{\text{1314,}}\,\,{\text{total:}}\,\,{\text{13,924)}}} \hfill  \\    {{\text{`no'  +  noun:}}} \hfill & {36\% } \hfill & {{\text{(sentence}}\,{\text{initial:}}\,\,{\text{1,501,496,}}\,\,{\text{total:}}\,\,{\text{ 4,133,238)}}} \hfill  \\    {{\text{`not}}\,{\text{all'  +  noun:}}} \hfill & {68\% } \hfill & {{\text{(sentence}}\,{\text{initial:}}\,\,{\text{8631,}}\,{\text{total:}}\,\,{\text{12,743)}}} \hfill  \\    {{\text{`some'  +  noun:}}} \hfill & {4.9\% } \hfill & {{\text{(sentence}}\,{\text{initial:}}\,\,{\text{3,19,453,}}\,\,{\text{total:}}\,\,{\text{6,462,706)}}} \hfill  \\    {{\text{`all'  +  noun:}}} \hfill & {5.7\% } \hfill & {{\text{(sentence}}\,{\text{initial:}}\,{\text{616,422,}}\,\,{\text{total:}}\,\,{\text{10,749,721)}}} \hfill  \\    {{\text{`many'  +  noun:}}} \hfill & {7.2\% } \hfill & {{\text{(sentence}}\,{\text{initial:}}\,\,{\text{420,016,}}\,\,{\text{total:}}\,\,{\text{5,873,004)}}} \hfill  \\    {{\text{`almost}}\,{\text{all'  +  noun:}}} \hfill & {19\% } \hfill & {{\text{(sentence}}\,{\text{initial:}}\,\,{\text{2663,}}\,\,{\text{total:}}\,\,{\text{14,338)}}} \hfill  \\   \end{array}$$

The diverging QEs in Experiment 1 (*få* ‘few’, and *inte alla* ‘not all’) have different sentence initial frequencies in the corpus findings (1.9% vs 68%) indicating that syntactic distribution is not the determining factor behind the results in Experiment 1. We also see that *några* (‘some’) has a slightly lower sentence initial frequency than the other positive QEs. However, the difference is very small, thus not clearly indicating that this is the reason for the lower ratings. Even though the corpus data also contains sentence initial non-subject QEs, we have no reason at this point to believe that this is the case to a significantly larger extent for the diverging QEs in Experiment 1, relative to the other QEs.
